# Clinical Characteristics and Outcomes in Heart Failure Patients with Implantable Pulmonary Artery Pressure Monitors: A Single Centre Irish Experience †

**DOI:** 10.3390/jcdd12010025

**Published:** 2025-01-14

**Authors:** Niall Leahy, Cillian O’Brien, Sara Essa Alsubai, Eileen Coen, Darragh Murphy, Faisal Sharif

**Affiliations:** 1 Department of Cardiology, University Hospital Galway, Saolta University Healthcare Group, Newcastle Road, H91YR71 Galway, Ireland; 2 Department of Medicine, University of Galway, University Road, H91TK33 Galway, Ireland

**Keywords:** heart failure, pulmonary artery pressure, remote monitoring, devices

## Abstract

**Background:** Hospitalisation for acute decompensated heart failure (HF) portends a poor prognosis. Fluid retention manifesting in dyspnoea and oedema are important clinical features of decompensated heart failure and drive hospital admissions. Intracardiac and pulmonary artery pressure (PAP) monitoring can help predict heart failure decompensation, as changes in these haemodynamics occur before clinical congestion manifests. **Methods:** A retrospective single centre analysis of patients who underwent insertion of the Cordella™ PA Sensor System (Endotronix, Inc., Chicago, IL, USA) in University Hospital Galway, Ireland, as part of three separate clinical trials—SIRONA 1, SIRONA 2, PROACTIVE HF, was performed. The primary clinical outcome assessed was the difference between HF hospitalisation pre- and post-sensor implantation. **Results:** In total, there were 33 patients with symptomatic HF who underwent device insertion between 2018 and 2023. All patients had NYHA class 3 heart failure, and 48.5% (*n* = 16) of patients had HF with reduced ejection fraction. Only one device-related complication was noted, and no pressure sensor failures occurred. In total, there were 26 admissions for HF decompensation 1-year pre-device insertion and only three admissions post-insertion. The difference in the mean number of HF hospitalisations per patient pre- and post-device insertion was 0.70 (*p* < 0.0001). The difference in mean NYHA class score pre- and post-insertion was 1.0 (*p* < 0.001). **Conclusions:** Data from this single-centre cohort study have shown that the insertion of the Cordella™ PA Sensor System in symptomatic HF patients was safe and resulted in statistically significant improvements in HF hospitalisations and NYHA class.

## 1. Introduction

Heart failure (HF) remains a prominent healthcare issue affecting a large proportion of the population worldwide [1]. It is associated with a significant burden of disability and hospital presentations, and exerts a considerable toll on national healthcare resources [2]. Its prevalence continues to rise due to our growing elderly population and improved survival rates following acute myocardial infarction [3]. Despite the improved outcomes that have been seen due to the evolution of the pharmacological management for patients with heart failure with reduced ejection fraction (HFrEF), hospitalisations secondary to HF still portends a grim prognosis for patients, with close to 50% requiring hospitalisation again in the next six months [1]. In Ireland, close to 100,000 people suffer from HF and this results in approximately EUR 280 million being spent per year in an attempt to manage this chronic condition [4].

The most common cause of hospitalisation in HF patients is HF decompensation [5]. This manifests as fluid retention resulting in clinical features which include lower limb oedema, dyspnoea, and orthopnoea. HF decompensation can lead to a progressive deterioration in overall myocardial function and quality of life [6]. Despite the often acute onset of symptoms of decompensation in patients, a rise in cardiac filling pressures occurs about four weeks prior to clinical decompensation [7]. Thus, attempting to maintain stability in HF patients solely through close monitoring of body weight and signs and symptoms of HF does not reliably improve clinical outcomes [2]. Pulmonary artery pressure (PAP) increases due to a rise in left atrial pressure in the setting of left ventricular dysfunction, and typically the PA pressures peak at the time of HF hospitalisation and decrease quickly following diuresis in hospital [1]. Therefore, the use of implantable PAP monitors has evolved as a method of remotely monitoring HF patients and adjusting their management accordingly, in an effort to improve their clinical outcomes. To date, implantable PAP monitors have been shown to reduce HF hospitalisations and improve quality of life in patients with symptomatic HF [8,9]. The current iteration of the European Society of Cardiology (ESC) guidelines on the diagnosis and treatment of heart failure recommend that their use should be considered in patients with symptomatic HF (class IIB recommendation) [10].

The use of pulmonary artery pressure monitors has arisen as part of an evolution in medicine in the use of cardiac implantable electronic devices (CIEDs) to enable remote continuous patient monitoring as part of disease management [11]. Remote monitoring using these devices has numerous potential advantages. This includes easier access for patients when specific devices, such as implantable cardiac defibrillators (ICDs), require periodic interrogation, as well as reduced hospital and clinic burden without compromising patient safety [12]. The use of CIEDs and remote monitoring extends to conditions other than heart failure, such as atrial fibrillation, where devices can facilitate the detection of atrial fibrillation and can be used to help monitor disease burden and progression [11].

The Cordella Heart Failure management system (the commercially available Cordella Heart Failure System (CHFS) and the investigational Cordella PA Sensor System (Endotronix Inc., Chicago, IL, USA)) is one of two implantable PAP monitors currently used in HF patients. It provides a variety of clinical information including pulmonary artery pressures, blood pressure, heart rate, and oxygen saturation, which allows clinicians to monitor the data to tailor patient management appropriately [13]. The safety and accuracy of the Cordella device have previously been demonstrated in the SIRONA-1 and SIRONA-2 trials [13,14]. The PROACTIVE-HF trial assessed the impact of managing seated mean PAP with the Cordella device on clinical outcomes in patients with HF. The results of this have recently been published and showed that the device safety profile was excellent, and there was 99.2% freedom from pressure sensor failure [15]. Additionally, the overall 6-month incidence of HF hospitalisations or all-cause mortality was low with PAP-guided HF management compared to the pre-defined performance goal, which was based on previous PAP-guided HF management trials, including GUIDE-HF and CHAMPION [15,16]. Additionally, clinically significant improvements in the Kansas City Cardiomyopathy Questionnaire (KCCQ) score, 6 min walk test, and New York Heart Association (NYHA) class were also seen [17].

University Hospital Galway has been one of the most prominent centres in Ireland in incorporating the use of the Cordella PAP monitor as part of the management of symptomatic HF patients. Patients have been enrolled from this site as part of the SIRONA-1, SIRONA-2, and PROACTIVE HF trials. The aim of this observational study was to examine the clinical efficacy and safety outcomes following the insertion of the Cordella PAP monitor in symptomatic HF patients in a single centre.

## 2. Materials and Methods

### 2.1. Population

Between December 2018 and March 2023, a total of 33 patients with NYHA class 3 heart failure underwent insertion of the Cordella PA Sensor System in University Hospital Galway, Ireland, and University of Galway, Ireland, as part of the SIRONA-1, SIRONA-2, and PROACTIVE-HF clinical trials. These clinical trials were sponsored by the device company. Eligible patients had to have at least one HF-related hospitalisation or HF treatment in a hospital setting, or unplanned outpatient clinic HF visit within the 12 months prior to enrolment. Patients were educated on how to use the device and written consent prior to inclusion in the trials was obtained. The Cordella sensor™ was placed in the right pulmonary artery of all patients via femoral vein access. The right pulmonary artery placement of the sensor allowed PAP measurements to be taken by patients using the handheld patient reader placed on the right side of the chest near the PAP monitor for 18 s. In addition to invasive PAP readings, other non-invasive clinical information inputted by the patient into the Cordella tablet including vital signs such as heart rate, blood pressure, oxygen saturation and body weight were securely transmitted to a data analysis platform [13].

### 2.2. Patient Follow-Up

Patients were advised to submit daily invasive PAP and non-invasive readings, which allowed for the calculation of mean PAP and enabled observation of the trend in values over time. This then permitted the patient’s clinician to remotely interrogate the data on the web-based patient management portal, make informed decisions on the patients’ HF management accordingly, and optimise their guideline-directed medical therapy (GDMT). Data transmitted by patients, which highlighted an upward trend in pulmonary artery pressures, were monitored by the clinical team. Data indicating this trend were actioned by the clinical team by analysing the changes in PAP, in addition to analysis of patients’ vital signs, such as heart rate, blood pressure, and body weight. Following this, a decision on patient management based on the available data was carried out. Management in the scenario of rising PA pressures indicating likely impending HF decompensation typically involved uptitration of maintenance loop diuretic dose for a specified duration, as well as optimisation of HF guideline-directed medical therapy. Prompt follow-up review, either remotely or in-person, following changes to pharmacological management, was performed by the study team. Patients were reviewed in person at one, three-, and six-months post-device implantation and then at six monthly intervals.

### 2.3. Outcomes

Electronic medical records were reviewed to determine the number of HF hospitalisations during the year before and year after wireless PAP implantation. Additional clinical outcomes recorded included NYHA class, N terminal pro-B-type natriuretic peptide (NT-proBNP), and 6 min walk test score, which were recorded at baseline and then at one, three-, and six-months post-implantation, followed then by six monthly intervals. In total, 4 patients did not have 12-month outcome data. Three of these patients died prior to reaching the one-year follow-up point, and one patient elected to discontinue the study. Their outcomes at time of last follow-up compared to baseline were therefore analysed. Patient-reported outcomes included KCCQ score, which was also reported at similar intervals to the clinical outcomes. A 6 min walk test was not assessed in SIRONA 1 patients, and thus, this outcome was compared between patients in SIRONA 2 and PROACTIVE-HF. Medications, specifically HF guideline-directed medical therapy (GDMT) and loop diuretic use at time of Cordella device insertion and at 1-year post-insertion, were analysed. The number of changes made to loop diuretics and each of the four classes of HF GDMT (beta blocker, angiotensin-converting enzyme inhibitor (ACEi)/angiotensin receptor blocker(ARB)/angiotensin receptor neprilysin inhibitor (ARNI), sodium–glucose co-transporter 2 inhibitor (SGLT2i), mineralocorticoid receptor antagonist (MRA)) over the one-year period was recorded.

### 2.4. Statistical Analysis

Descriptive statistics were used to evaluate baseline clinical and demographic characteristics. Results were reported as mean +/− standard deviation for continuous variables and counts and percentages were used for binary variables. Comparisons between baseline and one-year results for HF hospitalisations, NYHA class, 6MWT, and KCCQ score were performed and used a two-sample Student’s *t*-test. A two-sided *p* value < 0.05 was considered significant.

## 3. Results

Of the 33 patients who underwent implantable PAP monitor insertion, all had successful implantation. No device pressure sensor failure occurred, and only one device-related complication was noted. The mean age at the time of device insertion was 72 years (+/−10). In total, 30.3% (*n* = 10) of patients were female, 48.5% (*n* = 16) of patients had HF with reduced ejection fraction (HFrEF), and 15.2% (*n* = 5) had heart failure with mildly reduced ejection fraction (HFmrEF). In total, 54.5% (*n* = 18) of patients had co-existing coronary artery disease, and 36.4% (*n* = 12) were diabetics. A full breakdown of the baseline characteristics of the patients is outlined in Table 1.

**Figure 1:**
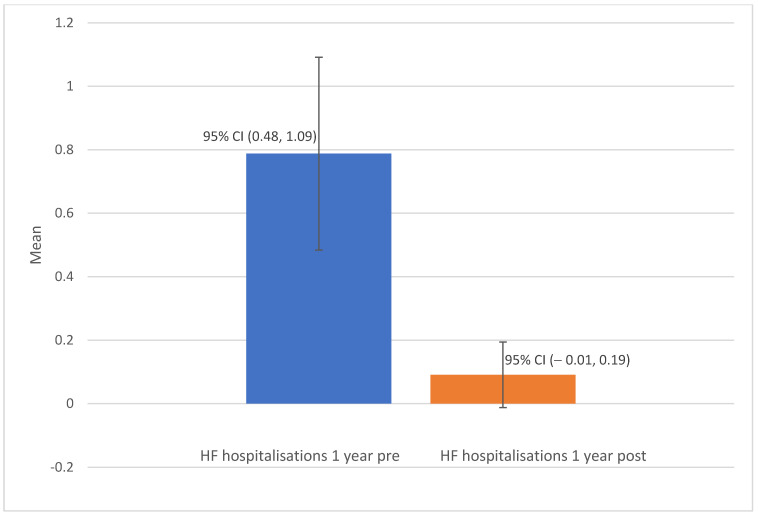
Barplot of mean HF hospitalisations pre- and post-PAP monitor insertion.

**Figure 2:**
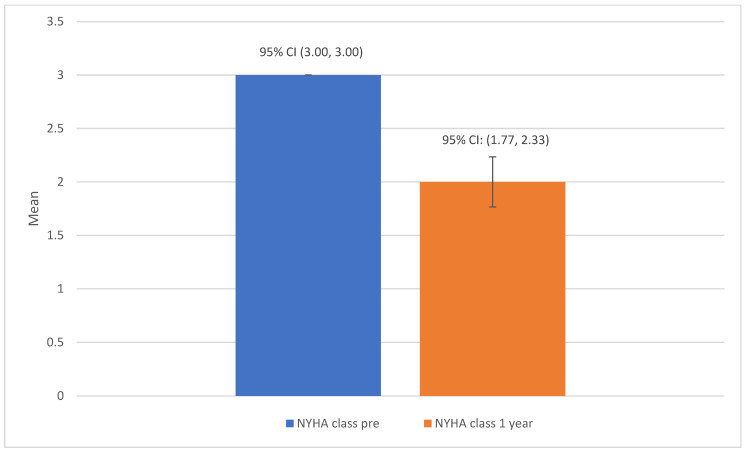
Barplot of mean NYHA class pre- and post-PAP monitor insertion.

The clinical measures at baseline versus one-year post-insertion of the Cordella device are shown in Table 2. In total, there were 26 admissions for HF decompensation across the group of 33 patients in the one-year pre-device insertion, and only 3 admissions were recorded for the same group in the one-year post-device insertion. The mean number of HF hospitalisations per patient one year pre- and post-device insertion was 0.79 (+/−0.86) and 0.09 (+/−0.29), respectively (*p* < 0.0001) (Figure 1). The difference in mean NYHA class score pre- and one-year post-implantable PAP monitor insertion was 1.0 (*p* < 0.001) (Figure 2). The mean 6 min walk test distance (6MWT) (metres) pre- and one-year post-device insertion was 259.56 (+/−170.81) and 291.04 (+/−128.52) metres, respectively (*p* = 0.327). The mean NT-proBNP level at baseline and one-year post-device insertion was 2138 ng/L (+/−2342) versus 2220 (+/−2782), with these values both skewed significantly by outlier values (*p* = 0.337). The mean baseline PA pressure, as measured by right heart catheterisation at the time of Cordella implant, was 28.79 mmHg (+/−12), and the mean PA pressure as measured by the Cordella device at 12 months post-insertion was 29.30 mmHg (+/−11). There was no statistically significant difference in PA pressures at one-year post-device insertion compared to baseline (*p* = 0.735). The mean overall summary KCCQ score pre-device insertion was 62.61 versus a mean score of 65.49 1-year post-device insertion (*p* = 0.427).

**Figure 3:**
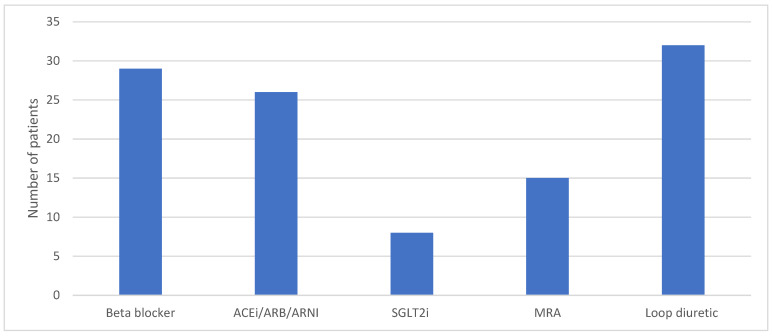
Barchart illustrating use of diuretics and HF GDMT at time of Cordella PAP monitor insertion.

**Figure 4:**
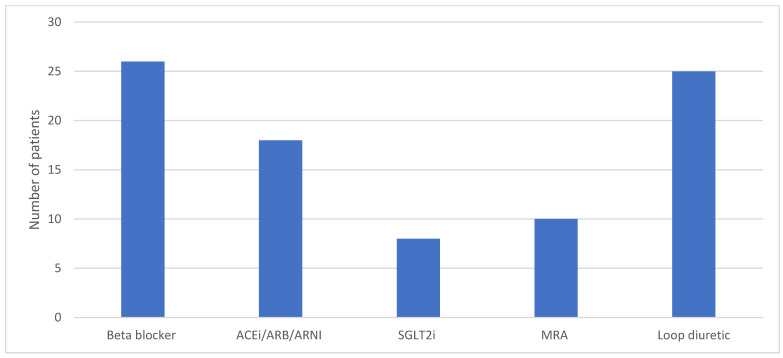
Barchart illustrating use of diuretics and HF GDMT at 12 months post-Cordella PAP monitor insertion.

Baseline use of loop diuretics and HF GDMT are illustrated in Figure 3. A significant proportion of patients were on a beta-blocker (*n* = 29/33), ACEi/ARB/ARNI (*n* = 26/33), and diuretic (*n* = 32/33) at the time of PAP monitor insertion. The use of SGLT2i (*n* = 8/33) and MRA (*n* = 15/33) was modest in comparison. The proportion of patients on these classes of medications at 12 months is outlined in Figure 4.

**Figure 5:**
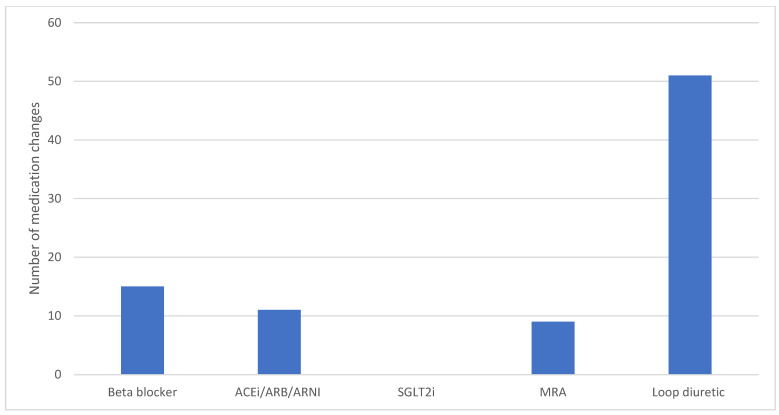
Barchart illustrating the changes in medication class made in the 12 months post-device insertion.

The changes in the use of diuretics and HF GDMT over the 12-month period following insertion of the PAP monitor were analysed (Figure 5). In total, 111 changes for 33 patients over the 12-month period were performed. Loop diuretics were the most commonly changed medication class, with 51 changes made in total over this period.

## 4. Discussion

In this study, we analysed data from a single centre in Ireland relating to the use of the Cordella PA Sensor System. To the best of the authors’ knowledge, this is the first reported cohort experience focusing solely on this specific PAP monitor in a European healthcare setting.

The pathophysiological basis upon which PAP monitors including Cordella work, relates to the known pattern of rising cardiac filling pressures a few weeks prior to the development of HF symptoms and signs. The ability to detect these changes in pressures in a timely fashion through remote monitoring offers a window of opportunity with which to modify HF treatment to prevent clinical congestion and subsequent hospitalisation [18]. Their potential role in the management of chronic HF patients is therefore easily apparent. With regards to advanced heart failure and patients requiring evaluation for a heart transplant, left ventricular assist device (LVAD), or palliative therapies, the role of PAP monitors is less clear. Patients in this category have previously been excluded from trials evaluating invasive pressure monitoring due to their poor prognosis. In patients approaching this stage of heart failure, characterised by high NYHA class, recurrent hospitalisations and high NT-proBNP levels, remote monitoring of PAP can enable aggressive uptitration of medications to help reduce pressures. This may help identify the appropriate timing for possible transplant and thus may offer an additional role for this technology [18].

The baseline characteristics of the patients in this study showed that over two-thirds of them were male and the mean age of patients was 72 years. Approximately half of the patients (*n* = 16) had known reduced left ventricular ejection fraction (LVEF) (LVEF < 40%) on their most recent transthoracic echocardiogram study. One-third of patients (*n* = 11) had preserved LVEF (LVEF > 50%). Previous randomised controlled trials involving PAP monitors have included HF patients irrespective of the baseline LVEF. Meta-analysis studies have previously demonstrated that the benefit observed from PAP monitor use with respect to HF hospitalisations remained consistent among patients with reduced and preserved LVEF [19].

This is one of the first studies to date analysing data from a single centre pertaining specifically to the use of the Cordella PA Sensor System in patients with symptomatic heart failure. The implantation of the device was shown to be safe in our cohort, with only one device-related complication noted, and no pressure sensor failures occurred. The results of our study provide interesting insights into the impact of the Cordella PA Sensor System on clinical outcomes following implantation. There have been modest observational data to date from centres on the specific use of the Cordella device in HF patients. Most studies to date assessing the impact of ambulatory PAP monitoring on outcomes in HF patients have involved the use of an alternative implantable PAP monitor—the CardioMEMS™ HF system (Abbott, Sylmar, CA, USA). These studies serve as a source of comparison with the results from our study. Dauw et al. performed a multicentre observational study, which included patients who received a PAP sensor from a site in Switzerland and the United Kingdom. Forty-eight patients in total were included, and nineteen of them received the Cordella device. Clinical outcomes were assessed as a whole rather than assessing them based on the specific type of PAP monitor inserted. A reduction in HF hospitalisations was seen at 6 and 12 months, and no device or system-related complications were observed [20].

The CardioMEMS European Monitoring Study for Heart Failure (MEMS-HF) study was a prospective non-randomised multicentre study evaluating the safety, feasibility, and performance CardioMEMS™ HF system (Abbott, Sylmar, CA, USA) over a 12-month follow-up period in Germany (26 centres), the Netherlands (4 centres) and Ireland (1 centre). It included 234 NYHA III HF patients who had a prior heart failure hospitalisation in the preceding 12 months [21]. This study demonstrated that 83 patients (35.5%) had a reduction in their NYHA class at 12 months. In our study, a smaller cohort of patients was evaluated but there was a significant reduction in mean NYHA class from 3 to 2 seen and overall, 26 patients (78%) had a reduction in their NYHA class.

A significant reduction in the mean number of hospitalisations was demonstrated post-Cordella insertion in our study. This has important prognostic significance, as we know that heart failure hospitalisation is a strong predictor for hospital readmission [22]. Our study showed that the mean number of hospitalisations per patient in the 12 months before and at 12 months post-Cordella insertion was 0.79 and 0.09, respectively, which corresponds to an 88% reduction (*p* < 0.001). This suggests that the Cordella device can impact meaningfully on a critical heart failure clinical outcome and compares favourably to other observational data we have from studies examining this outcome in patients who have undergone CardioMEMs device insertion. Gibson et al. assessed outcomes in HF patients who underwent implantation of the CardioMEMs device in a single centre in Canada and found an 87% reduction in HF hospitalisations in the 12 months post-insertion [23]. The COAST study, which was a multicentre study that assessed clinical outcomes in patients post-CardioMEMS device insertion, similarly found a greater than 80% reduction in HF hospitalisations at one year [24]. The aforementioned MEMS-HF trial reported a 62% reduction in HF hospitalisations in the one-year post-device insertion [21]. Codina et al. performed a single-centre study in Spain involving HF patients who had undergone CardioMEMS implantation. Two-thirds of the patients had NYHA class III HF. They found a 78% reduction in the rate of HF hospitalisations at 1-year post-insertion, which was statistically significant [5]. This latter study focusing on a single centre in Spain has additional relevance as the investigators performed a cost–benefit analysis and estimated a reduction in healthcare costs with the PAP monitor compared to usual care by the end of the second year post-insertion [5]. Spain has a healthcare system that is predominantly funded by public means, and thus when considering incorporating new technologies like the use of PAP monitors for remote management of HF patients, demonstrating evidence of its efficacy and economic value is important. Similar to Spain, Ireland is actively seeking to implement a universal health and social care system as part of the Sláintecare health strategy set out by the Irish Government in 2017 [25]. Our single centre data on the use of remote PAP monitoring in HF patients showed a significant reduction in a key heart failure outcome—HF hospitalisations, and we would envisage that cost-effectiveness would be demonstrated if an analysis, similar to the one performed by Codina et al., was performed.

A reduction in functional ability and exercise tolerance in patients with HF is associated with a worse overall prognosis and a reduction in quality of life. The 6MWT is a widely available and well-tolerated test that is frequently used to assess HF patients’ functional capacity [26]. In our study, numerical improvements were seen in 6MWT scores for patients post-Cordella insertion compared to pre-device insertion. The mean score (metres) pre- and 12 months post-device insertion was 259.56 +/− 170.81 and 291.04 +/− 128.52, respectively (*p* = 0.327).

The mean NT-proBNP level (ng/L) pre- and post-device insertion was 2138 +/− 2342 and 2220 +/− 2782, respectively (*p* = 0.876). There was therefore no statistically significant changes in NT-proBNP level, although outlier values likely affected this considerably, and thus this limits the degree of meaningful interpretation.

Mean PAP also did not change significantly post-Cordella insertion, which was surprising. Mean PAP pre- and 12 months post-device insertion were 28.79 +/− 12 and 29.30 +/− 11, respectively (*p* = 0.735). Other studies assessing the use of PAP monitors in HF patients have previously shown significant improvements in PAP post-insertion [21,23,24].

KCCQ score is a validated health status measure for patients with HF and an important patient-related outcome measure when evaluating the merits of an intervention aimed at HF patients [27]. There was a small improvement in KCCQ overall summary scores seen post-Cordella device insertion in our patient cohort. The mean score pre-device insertion was 62.61 +/− 22.72 and at one year (or time of last follow-up) post-insertion, the mean score was 65.49 +/− 24.63 (*p* = 0.427).

The majority of medication changes over the one-year period post-Cordella insertion involved loop diuretics, with no significant increase seen in the use of HF GDMT. While tailoring diuretic regimen based on the interpretation of data provided by Cordella was likely the main driver of the reduced rates of HF hospitalisation, the optimisation of HF GDMT was disappointing in comparison. STRONG-HF has previously demonstrated the benefits of high-intensity uptitration of HF GDMT, irrespective of baseline LVEF, and thus the information provided by remote PAP monitoring should act as a tool to help optimise HF GDMT use in the future [28].

The ESC provides a strong recommendation for the use of remote monitoring as a standard way to perform periodic checks of CIEDs, and a discussion point associated with this topic is the issue of reimbursement. Currently, there is considerable heterogeneity in Europe with regard to reimbursement for remote monitoring of CIEDs [29]. In Ireland, remote monitoring is currently not approved for reimbursement. With growing interest in this field of medicine, the landscape will likely continue to change, and reimbursement for remote monitoring may be implemented on a wider scale in the future in an effort to promote its use.

There were several limitations of our study, which are important to acknowledge. First, the observational nature of our study means that the clinical outcomes may have been influenced by confounding factors. Second, the overall number of patients included in our study was reasonably small and thus limits the generalisability of the results. Nevertheless, in terms of patient numbers, it compares similarly to the other single-centre studies, which have been discussed herein. Third, four of the patients did not reach the 12-month follow-up point, and therefore, the time of the last follow-up was chosen as the point to assess clinical outcomes in these patients. Lastly, mean PAP was assessed at a single point at 12 months post-insertion (or time of last follow-up). Assessing the temporal trends in PAP from the time of insertion, as opposed to one point in time post-insertion, may have provided more meaningful insight for this outcome measure, given the association between high PAP and HF hospitalisations [30].

## 5. Conclusions

In this single-centre Irish real-world experience study, the use of the Cordella™ PA Sensor System in symptomatic HF patients was shown to be safe and effective in improving clinical outcomes [31]. Statistically significant improvements in HF hospitalisations and NYHA class were seen in patients following the implantation of this remote PAP monitor device. Future studies with larger patient cohorts and longer follow-up duration, as well as cost–benefit analyses, will be important to help further define the position that this technology has in the HF management algorithm.

## Figures and Tables

**Table 1:** Baseline characteristics of patients undergoing implantable pulmonary artery pressure monitor insertion.

Characteristic	N = 33	Percentage (%)
Male	23	70
Female	10	30
Age ^1^—years	72 (+/−10)	
Hyperlipidaemia	14	42
Diabetes mellitus	12	36
Hypertension	23	70
Coronary artery disease	18	55
Previous myocardial infarction	14	42
Atrial fibrillation/flutter	21	64
Chronic kidney disease (eGFR < 60)	23	70
Previous stroke/transient ischemic attack (TIA)	4	12
Peripheral vascular disease	3	9
NT-proBNP ^1^ ng/L	2138 (+/−2342)	
NYHA class (>/=3)	33	100
Left ventricular ejection fraction:		
<40	16	48
40–50%	5	15
>50%	11	33
Mean PAP > 20 mmHg (based on right heart catheterisation assessment)	24	73

^1^ Mean (+/−SD); eGFR—estimated glomerular filtration rate (mL/min/1.73 m^2^); NT-proBNP—N-terminal prohormone of brain natriuretic peptide; NYHA—New York Heart Association; PAP—pulmonary artery pressure.

**Table 2:** Clinical measures at baseline versus one-year post-insertion of Cordella device.

Variable	Baseline	One-Year Post-Insertion (or Last Follow-Up) of the Cordella Device	*p* Value
NYHA class	3 +/− 0	2 +/− 0.661	<0.001
6MWT (m)	259.56 +/− 170.81	291.04 +/− 128.52	0.327
NT-proBNP (ng/L)	2138 +/− 2342	2220 +/− 2782	0.876
Mean PAP (mmHg)	28.79 +/− 11.75	29.30 +/− 10.76	0.735
KCCQ overall summary score	62.61 +/− 22.72	65.49 +/− 24.63	0.427

Values stated other than *p* values are mean values +/−standard deviation. 6MWT—6 min walk test; KCCQ—Kansas City Cardiomyopathy Questionnaire.

## Data Availability

Restrictions apply to the availability of these data. Data were obtained from Endotronix and are available with the permission of Endotronix.
